# The Yellow Fever

**Published:** 1905-08

**Authors:** 


### EDITORIAL NOTES AND COMMENTS.

The Business office of THE JOURNAL-RECORD is No. 1007-1008 Century Building.

The Editorial office is 318-319-320 The Prudential.

Address all Business communications to Dr. M. B. Hutchins, Mgr.

Make remittances payable to THE ATLANTA JOURNAL-RECORD OF MEDICINE.

On matters pertaining to the Editorial and Original Communications address Dr Bernard Wolff, Atlanta.

Reprints of original articles will be furnished at cost price. Requests for the same should always be made *on the manuscript*.

We will present, post-paid, on request, to each contributor of an original article, twenty (20) marked copies of THE JOURNAL-RECORD containing such article.

### THE YELLOW FEVER.

The report of the occurrence of several cases of yellow fever in New Orleans has sent a wave of alarm throughout the country and occasioned the establishment of quarantine against the infected districts by some of the border States.

There is perhaps no disease about which more accurate information of its mode of transmission is possessed than yellow fever. Such data furnish indications for combatting the spread of the disease that are so simple and clear as to quell the fear that there will ever again be serious epidemics of it, such as have occurred in the past, and upon which the general dread of the disease is founded. The definite knowledge of the etiology of yellow fever dates back scarcely five years. Yet in this brief space our conceptions of the disease, in many important essentials, have been completely altered and placed from the speculative into the proven.

This revolution was occasioned by the result of the labors of the late Major Walter Reed, of the Medical Corps of the United States army, and his asssociates in the island of Cuba. His history-making investigation may be appropriately described in the words of one of its participants, Dr. James Carroll. It is taken from an article by Dr. J. Salinger, in Modern Clinical Medicine. Dr. Carroll made the following statement:

"Early in 1900 yellow fever appeared among the American troops at Havana, and during the summer it became quite prevalent among the Americans and Spaniards in and about the city. In order to take advantage of the opportunity to continue the study of the etiology of the disease, a Board of Army Medical Officers was ordered to meet at Havana for the purpose. It was composed of Major Walter Reed, Surgeon U. S. Army, the writer, and Dr. Jesse M. Lazear, non-immunes, and Dr. Aristides Agramonte, a Cuban immune. The three last named were contract surgeons. Dr. Agramonte and Dr. Lazear were already on the island; Dr. Reed and the writer arrived at Havana, June 25, 1900, and within a day or two the board was organized and work begun. Cultures from the blood of eighteen patients drawn during life were carefully studied, and from these, as well as from cultures made at eleven autopsies, the board failed to recover bacillus icteroides; the conclusion was drawn, therefore, that the organism could be excluded from further consideration."

In 1881 Dr. Carlos Finlay proposed a theory that the transmission of the disease was due to the mosquito; this view was neglected for a long time, but with the development of our knowledge regarding the part played by the mosquito in the transmission of malarial fever and on account of many points of similarity between the two affections, malaria and yellow fever, such as the prevalence in regard to season, the zone in which it prevails, and the way in which extension of the affection occurs, the subject was taken up again. After much laborious work and by unobjectionable experiments it was determined that the mosquito, the stegomyia fasciata, transmits the disease. This was not only proven by the Commission of a Board of Army Medical Officers, which consisted of Dr. Reed, Dr. Lazear, Dr. Agramonte, and Dr. Carroll, but was also confirmed by the French Yellow Fever Commission previously referred to. Dr. Carroll * writes:

* *New York Medical Journal* and *Philadelphia Medical Journal*, February 6 and 13, 1904.

"On the afternoon of July 27, 1900, I subjected myself to the bite of an infected mosquito applied by Dr. Lazear. The insect which had been hatched and reared in the laboratory, had been caused to feed upon four cases of yellow fever, two of them severe, and two mild. The first patient, a severe case, was bitten twelve days before; the second, third and fourth patients had been bitten six, four, and two days previously, and these cases were in character, mild, severe, and mild, respectively. In writing to Dr. Reed that night of the incident, I remarked jokingly that if there were anything in the mosquito theory I should have a good dose. And so it happened. After having slight premonitory symptoms for two days I was taken sick on August 31st, and on September 1st, I was carried to the yellow fever camp. My life was in the balance for three days and my chart shows that on the fifth, sixth, and seventh days my urine contained eight-tenths and nine-tenths of moist albumin. The tests were made by Dr. Lazear. I mention this particularly because the result obtained in the case does not agree with the twentieth conclusion of Marchoux, Salimbeni, and Simond, that the longer the interval that elapses after the infection of the mosquito the more dangerous he becomes. Twelve days, the period above cited, is the shortest time in which the mosquito has been proven to be capable of conveying the infection. It is my opinion that the susceptibility of the individual bitten is a much more potent factor in determining the severity of the attack than the duration of the infection of the mosquito, or the number of mosquitoes applied. On the day that I was taken sick, August 31, 1900, Dr. Lazear applied the same mosquito, with three others, to another individual who suffered a comparatively mild attack and was well before I had left my bed. It so happened that I was the first person in whom the mosquito was proven to convey the disease."

Dr. Lazear of the American Commission and Dr. Myers of the Liverpool Commission both died of yellow fever from the bites of infected mosquitoes, thus two names must be added to the already large roll of martyrs of science.

Yellow fever is a disease of coast countries, rarely occurring in regions 1,500 feet above the sea level. The development of the disease is favored by filth, crowding of population, poor housing, hot season, and great humidity.

The view that the disease is transmitted by fomites has been almost entirely abandoned, owing to the experiments of the Yellow Fever Commission of the United States Army. There is great difference in the individual susceptibility to contract yellow fever; most authors agree that the negro race is less susceptible than the white race, this manifesting itself by the milder character of the disease in the negro than in the white. Males are more often attacked than females, and newcomers who are not acclimated usually acquire the disease readily. Guitèras ascribes the immunity of the natives in a yellow fever zone to the fact that they have passed through an attack of yellow fever in infancy or childhood, thus being protected by a subsequent immunity. He maintains that in tropical and subtropical regions in which yellow fever exists it is essentially an infection of childhood. The conclusions from the original results of the United States Army Yellow Fever Commission are as follows: *

1. *Bacillus icteroides*, Sanarelli, and the hog cholera bacillus are practically identical.

2. Yellow fever is transmitted by the mosquito *Stegomyia fasciata.*

3. This mosquito may convey the disease as early as on the twelfth day after biting the patient, and it may retain the power to do so as long as it lives.

4. Yellow fever can be transmitted by the hypodermic injection of blood drawn from a patient in the first, second, or fourth days of the disease.

5. Yellow fever is not communicated by fomites.

6. The infectious agent of yellow fever can be passed through a filter that is impermeable to ordinary bacteria.

7. The infectious property of blood drawn from yellow fever patients is destroyed by a temperature of 55° C., maintained for ten minutes.

The disease is not contagious and there appears to be but slight risk in those nursing yellow fever patients. The *period of incuba-*

* *New York Medical Journal* and *Philadelphia Medical Journal*, February 6 and 13, 1904.

*tion* varies greatly, in general it is from one to seven days, in some cases even a little longer than this.

So far as the local conditions are concerned though the stegomyia fasciata exists in and around Atlanta, the history of yellow fever in the past as far as concerns the conduct of cases imported from elsewhere indicates the existence of influences inimical to the spread of yellow fever. There has been no well-founded authenticated case of yellow fever originating here or transmitted to a sound from an infected person, though from time to time the disease has developed here in individuals inoculated in the fever zone. Whether or not this belief in local immunity is warranted by physical facts is a matter of opinion; it has at least not yet been shaken by any circumstance that tended to reflect upon its truth.

